# Association of active and passive smoking with noncommunicable diseases in a large health check-up population: A cross-sectional study in Changzhi, Shanxi province, China

**DOI:** 10.18332/tid/226557

**Published:** 2026-08-27

**Authors:** Zhiyong Dou, Ping Wu, Lujie Wang, Jing Guo

**Affiliations:** 1 Health Management Center, Heping Hospital, Changzhi Medical College, Changzhi, China; 2 Research Management Department, Heping Hospital, Changzhi Medical College, Changzhi, China

**Keywords:** tobacco smoking, secondhand smoke, non-communicable diseases, cross-sectional study

## Abstract

**INTRODUCTION:**

Tobacco smoke, encompassing both active smoking and secondhand smoke (SHS) exposure, constitutes a leading modifiable risk factor for the global burden of non-communicable diseases (NCDs). This study aimed to examine the association of active smoking and SHS with the presence of NCDs among health check-up populations, and to explore the dose-response association according to daily cigarette consumption and smoking duration.

**METHODS:**

A cross-sectional study was conducted among 38906 adults aged ≥18 years who were local residents undergoing physical examination in Changzhi, Shanxi Province from January to December 2025. Questionnaire interviews were used to capture the demographics, lifestyle habits, tobacco smoke status (active smoking, SHS exposure, daily cigarette consumption, and smoking duration), and clinically diagnosed NCDs. Multivariable logistic regression estimated the adjusted odds ratios (AORs) and 95% confidence intervals (CIs), with adjustment for potential confounders. In this study, a p<0.05 was considered statistically significant.

**RESULTS:**

The overall prevalence of current smoking was 27.3% (51.3% in men, 0.3% in women), and 26.2% of participants had at least one NCD. After full adjustment, active smoking remained independently associated with increased odds of NCDs (AOR=1.26; 95% CI: 1.18–1.35). A modest dose-response gradient was observed for smoking duration: compared with <5 years, the AORs were 1.24 (95% CI: 1.08–1.43) for 5–14 years, 1.23 (95% CI: 1.09–1.39) for 15–24 years, and 1.37 (95% CI: 1.19–1.58) for ≥25 years. Daily consumption of ≥20 cigarettes was modestly associated with elevated risk (AOR=1.13; 95% CI: 1.03– 1.25). Regarding SHS, the crude association was significant (OR=1.25; 95% CI: 1.16–1.35), but attenuated after adjusting for potential confounders (AOR=1.04; 95% CI: 0.95–1.13). In subgroup analyses, active smoking was associated with hypertension, diabetes, cardiovascular disease and dyslipidemia, and the doseresponse relationships were also observed for smoking duration.

**CONCLUSIONS:**

Active smoking independently increases the odds of prevalent NCDs, especially among long-term smokers. The attenuated effect of SHS highlights the challenge of isolating its independent role in observational settings. So comprehensive tobacco control strategies and targeted cessation interventions for long-term smokers are important.

## INTRODUCTION

Non-communicable diseases (NCDs), principally cardiovascular diseases, diabetes mellitus, chronic respiratory diseases, and cancer, have surpassed communicable diseases as the leading cause of morbidity and mortality worldwide, accounting for approximately 74% of all deaths globally^1^. In China, the epidemiological transition has been particularly pronounced, with NCDs contributing to over 85% of total mortality and posing an immense burden on the healthcare system^2^. Major behavioral risk factors – including tobacco use, unhealthy diet, physical inactivity, and harmful alcohol consumption – drive this epidemic, and their modification has become a cornerstone of public health policy^3^.

Among these risk factors, tobacco smoking stands out as the single most preventable cause of death. Globally, there are over 1.3 billion tobacco users, and smoking-attributable mortality exceeds 8 million individuals annually^4^. The pathophysiological pathways by which active smoking promotes NCDs are well established: the thousands of toxicants and reactive oxygen species in tobacco smoke trigger oxidative stress, systemic inflammation, and insulin resistance, thereby accelerating atherosclerosis, oncogenesis, and metabolic derangements^5,6^. Large-scale cohort studies and meta-analyses have consistently demonstrated that current smokers face a 2- to 4-fold increased risk of coronary heart disease, stroke, type 2 diabetes, and dyslipidemia compared with never smokers, with risk escalating according to both daily consumption and cumulative pack-years^7,8^. In China, where male smoking prevalence remains high at approximately 50% while female smoking is rare (<3%), the gendered pattern of tobacco-attributable NCDs deserves special attention^9^.

Beyond active tobacco smoking, exposure to secondhand (SHS) has been causally linked to cardiovascular and respiratory diseases^10,11^. The U.S. Surgeon General’s Report in 2006 concluded that there is no risk-free level of SHS exposure, and subsequent global estimates indicate that around one-third of adults are regularly exposed^12,13^. However, in observational studies, the independent effect of SHS on composite NCD outcomes often attenuates considerably after adjustment for confounding factors, as SHS exposure correlates with active smoking, socioeconomic status, and other unhealthy behaviors. Furthermore, in people with a high burden of household air pollution, disentangling the contributions of SHS and indoor biomass combustion to NCD risk becomes methodologically challenging^14^.

Previous investigations have predominantly examined individual NCDs in relation to smoking, whereas data on a composite NCD outcome incorporating multiple cardiometabolic conditions in a health check-up setting remain scarce^15^. Moreover, few studies have simultaneously adjusted for a comprehensive set of confounders, including sedentary behavior, sleep duration, and kitchen fume exposure, when analyzing both active and passive smoking^16^. Utilization of a large health examination database offers the advantage of standardized anthropometric measurements and systematic disease ascertainment, providing a robust platform to quantify these associations. Therefore, this study aimed to understand the prevalence of tobacco smoke exposure in a large sample of individuals undergoing physical examinations and to examine the association of active smoking and SHS with the presence of NCDs, and to explore the dose-response gradients according to daily cigarette consumption and smoking duration.

## METHODS

### Study population

This cross-sectional study was conducted among adults (≥18 years) who participated in a health examination program in Changzhi City, Shanxi Province, China, during January and December 2025. In this study, we applied the sample size calculation formula n = [μ_α_^2^ × p(1-p)]/δ^2^ and set p for smoking prevalence as 30%, α=0.05, δ=2% of p, and a non-response rate of 10%. The sample size calculation indicated that at least 24900 adults should be recruited. After excluding records with missing key variables, 38906 participants (20581 men, 18325 women) were eventually included and analyzed. This study was reviewed and approved by the Heping Hospital, Changzhi Medical College (2026-098), and all participants provided written informed consent. This study adhered to the Declaration of Helsinki.

### Diagnosis and enrollment

Hypertension, diabetes, cardiovascular disease, and dyslipidemia were ascertained from participants’ healthcare records or physical examination findings, and diagnoses were made in line with the Chinese clinical practice guidelines for the respective chronic diseases. The inclusion criteria were: 1) aged ≥18 years, and 2) local residence in Changzhi city undergoing physical examination. The exclusion criteria included: 1) inability or refusal to provide informed consent, and 2) presence of severe neurological or psychiatric disorders that might interfere with communication or cooperation.

### Data collection

Data were collected through face-to-face interviews and physical examinations conducted by the general practitioners in Heping Hospital affiliated to Changzhi Medical College. The questionnaire includes: 1) demographic characteristics (age, gender, education level, individual income, residency status, marital status, occupation, etc.); 2) history of 4 types of noncommunicable diseases (hypertension, type 2 diabetes mellitus, cardiovascular disease, and dyslipidemia); and 3) tobacco consumption information (age of tobacco smoking initiation, smoking duration and daily tobacco consumption, etc.).

### Definition and classification

A smoker was defined as a person who smoked ≥100 cigarettes in a lifetime. Among smokers, daily cigarette consumption was dichotomized as <20 versus ≥20 cigarettes/day, and years of smoking was categorized as <5, 5–14, 15–24, and ≥25 years. SHS exposure was defined as self-reported regular (≥3 times/week, ≥15 min per occasion) passive inhalation of smoke from others in indoor or working environments. Furthermore, the primary outcome was the presence of any NCD, defined as having at least one of the following physician-diagnosed conditions documented in the health examination record: hypertension, diabetes mellitus, cardiovascular disease (including coronary heart disease and stroke), and dyslipidemia. The number of coexisting NCDs (0, 1, ≥2) was also recorded.

Demographic characteristics were categorized to facilitate analysis. Age was stratified into six groups: <35, 35–44, 45–54, 55–64, 65–74, and ≥75 years. Education level was classified as primary school or lower, junior high school, senior high school, and college or higher. Marital status was categorized as unmarried, married, and divorced/other. Body mass index (BMI, kg/m^2^) was classified into <23.9 (underweight/normal), 24.0–28.0 (overweight), and >28.0 (obese). Daily sleep time (<6 h vs >6 h) and daily sedentary time (>5 h vs <5 h) were dichotomized, respectively. Alcohol drinking (Yes, No), kitchen fume exposure (regular exposure to cooking oil fumes: Yes, No), and anthropometric measurements, including waist and hip circumference, were obtained by trained staff.

### Statistical analysis

Data were analyzed using SPSS 22.0 (IBM Corp., USA). Quantitative variables were expressed as mean and standard deviation (SD) or as median and interquartile range (IQR) as appropriate, and t-tests and non-parametric rank sum tests (Mann-Whitney U test) were used for comparisons between groups. Qualitative variables were expressed as frequencies (n) and percentages (%), and the chi-squared test was used for comparison between groups. In this study, multivariable logistic regression models were constructed to estimate adjusted odds ratios (AORs) and 95% confidence intervals (CIs) for the association between tobacco smoke variables and NCDs. Three models were used: Model A was unadjusted; Model B adjusted for age, gender, and BMI; and Model C further adjusted for education level, marital status, alcohol drinking, daily sleep time, daily sedentary time, and kitchen fume exposure. The overall NCD outcome was analyzed in all models, while for individual NCD components (hypertension, type 2 diabetes mellitus, cardiovascular disease, and dyslipidemia), unadjusted ORs were computed for subgroup purposes. All statistical tests were two-tailed and a p<0.05 was considered statistically significant.

## RESULTS

The mean age of the 38906 participants was 48.2 years (SD=13.2), with males being slightly older than females (48.6 vs 47.7 years, p<0.001). The age distribution revealed that the majority of participants fell within the 35–54 years age range, accounting for 50.2%. Males had significantly higher mean BMI (25.9 vs 23.7 kg/m^2^), leading to a substantially higher prevalence of being overweight (48.4% vs 32.6%) and obese (24.4% vs 9.6%) compared to females (all p<0.001). The overall prevalence of NCDs was markedly higher in men than in women (32.5% vs 19.2%), with hypertension (24.3% vs 12.8%), diabetes (8.6% vs 3.9%), cardiovascular disease (1.1% vs 0.4%), and dyslipidemia (2.3% vs 1.8%) all demonstrating significant gender disparities (p<0.001). Regarding behavioral characteristics, 51.5% of men reported current alcohol consumption, while only 0.7% of women did. The proportion of individuals sleeping <6 hours daily was slightly higher among men (18.3% vs 14.8%), and daily sedentary time exceeding 5 hours was marginally more frequent in men (51.0% vs 49.0%). A striking gender difference emerged for kitchen fume exposure, which was reported by only 0.6% of males but 7.7% of females (Table 1).

**Table 1:** Demographic, lifestyle and clinical characteristics of residents undergoing physical examination in Changzhi, Shanxi Province, China, January–December 2025 (N=38906)

*Variables*	*Male (N=20581)* *n (%)*	*Female (N=18325)* *n (%)*	*Total (N=38906)* *n (%)*	*p*
**Age** (years)				0.000
<35	3359 (16.3)	3102 (16.9)	6461 (16.6)	
35–44	5121 (24.9)	4769 (26.0)	9890 (25.4)	
45–54	4995 (24.3)	4652 (25.4)	9647 (24.8)	
55–64	4516 (21.9)	3881 (21.2)	8397 (21.6)	
65–74	2037 (9.9)	1550 (8.5)	3587 (9.2)	
≥75	553 (2.7)	371 (2.0)	924 (2.4)	
Mean (SD)	48.6 (13.4)	47.7 (12.9)	48.2 (13.2)	0.000
**Education level**				0.560
Primary and lower	3046 (14.8)	2790 (15.2)	5836 (15.0)	
Junior high	7245 (35.2)	6373 (34.8)	13618 (35.0)	
Senior high	6195 (30.1)	5476 (29.9)	11671 (30.1)	
College and higher	4095 (19.9)	3686 (20.1)	7781 (19.9)	
**Marital status**				0.580
Married	14448 (70.2)	12786 (69.8)	27234 (70.0)	
Unmarried	4075 (19.8)	3706 (20.2)	7781 (20.0)	
Divorced/other	2058 (10.0)	1833 (10.0)	3891 (10.0)	
**BMI** (kg/m^2^)				0.000
<23.9 (low/normal)	5598 (27.2)	10603 (57.8)	16201 (41.6)	
24.0–28.0 (overweight)	9957 (48.4)	5970 (32.6)	15927 (40.9)	
>28.0 (obese)	5026 (24.4)	1752 (9.6)	6778 (9.6)	
Mean (SD)	25.9 (3.3)	23.7 (3.2)	24.9 (3.5)	0.000
**NCDs**	6693 (32.5)	3508 (19.2)	10201 (26.2)	0.000
Hypertension	4992 (24.3)	2341 (12.8)	7333 (18.8)	0.000
Diabetes	1778 (8.6)	717 (3.9)	2495 (6.4)	0.000
Cardiovascular disease	225 (1.1)	69 (0.4)	294 (0.8)	0.000
Dyslipidemia	470 (2.3)	324 (1.8)	794 (2.0)	0.000
**Waist circumference**, mean (SD)	91.5 (8.7)	79.6 (8.8)	85.7 (10.6)	0.000
**Hip circumference**, mean (SD)	100.3 (6.9)	95.5 (7.1)	97.9 (7.4)	0.000
**Waist-hip ratio**, mean (SD)	0.9 (0.3)	0.8 (0.1)	0.9 (0.2)	0.000
**Alcohol drinking**	10607 (51.5)	126 (0.7)	10733 (27.6)	0.000
**Daily sleep time <6 h**	3767 (18.3)	2710 (14.8)	6477 (16.6)	0.000
**Daily sedative time >5 h**	3499 (51.0)	3361 (49.0)	6860 (17.6)	0.001
**Kitchen fume exposure**	122 (0.6)	1415 (7.7)	1537 (4.0)	0.000

BMI: body mass index. NCD: non-communicable disease.

### Tobacco smoking status and NCD prevalence

**Figure 1:**
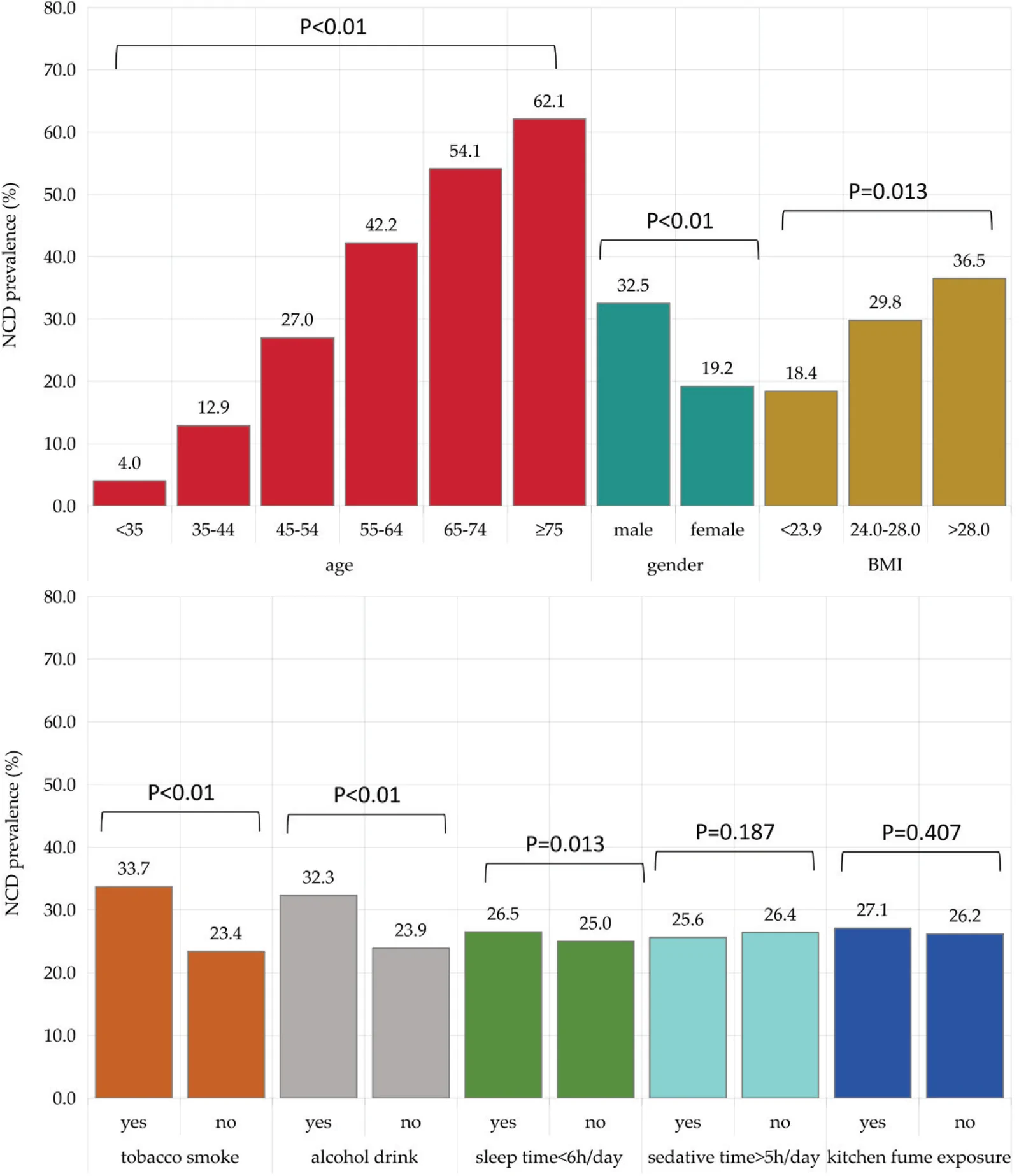
The prevalence of NCDs among residents undergoing physical examination by different age, gender, BMI, tobacco smoking status, alcohol drinking status, and different sleep, sedative life and kitchen fume exposure habits in Changzhi, Shanxi Province, China, January–December 2025 (N=38906)

In Table 2, the overall prevalence of active smoking was 27.3%, with 51.3% for males and only 0.3% for females. Among active smokers, the majority (73.7%) consumed <20 cigarettes per day; the distribution of smoking duration revealed that 17.1% had smoked for <5 years, 39.3% for 5–14 years, 26.7% for 15–24 years, and 16.9% for ≥25 years. Secondhand smoke exposure was reported by 9.1% of participants, with men more frequently affected (15.9%) than women (1.6%). The overall prevalence of any NCD was 26.2%, with 22.4% of participants having a single NCD and 3.8% having ≥2 conditions. Furthermore, the prevalence of NCDs was higher in older participants, with 4.0% for <35 years, 12.9% for 35–44 years, 27.0% for 45–54 years, 42.2% for 55–64 years, 54.1% for 65–74 years, and 62.1% for ≥75 years. Male participants had higher NCD prevalence, and participants with higher BMI also had more NCD problems, which were 29.8% for overweight (BMI: 24.0–28.0) and 36.5% for obese (BMI: >28.0). Participants with tobacco smoking (33.7% vs 23.4%), alcohol drinking (32.3% vs 23.9%), and daily sleep time <6 hours (26.5% vs 25.0%) also had a higher prevalence of NCDs (Table 2 and Figure 1).

**Table 2:** The tobacco smoke and NCD prevalence among residents undergoing physical examination in Changzhi, Shanxi Province, China, January–Decemeber 2025 (N=38906)

*Variables*	*Male (N=20581)* *n (%)*	*Female (N=18325)* *n (%)*	*Total (N=38906)* *n (%)*	*p*
**Tobacco smoking**				0.000
Yes	10556 (51.3)	51 (0.3)	10607 (27.3)	
No	10025 (48.7)	18274 (99.7)	28299 (72.7)	
**Daily cigarette consumption**				0.007
<20	7773 (73.6)	46 (90.2)	7819 (73.7)	
≥20	2783 (26.4)	5 (9.8)	2788 (26.3)	
**Years of tobacco smoking**				0.000
<5	1781 (16.9)	36 (70.6)	1817 (17.1)	
5–14	4159 (39.4)	7 (13.7)	4166 (39.3)	
15–24	2825 (26.8)	6 (11.8)	2831 (26.7)	
≥25	1791 (17.0)	2 (3.9)	1793 (16.9)	
**Secondhand smoke exposure**				0.000
Yes	3272 (15.9)	285 (1.6)	3557 (9.1)	
No	17309 (84.1)	18040 (98.4)	35349 (90.9)	
**NCDs**				0.000
Yes	6693 (32.5)	3512 (19.2)	10205 (26.2)	
No	13888 (67.5)	14813 (80.8)	28701 (73.8)	
**Number of NCDs**				0.000
0	13888 (67.5)	14813 (80.8)	28701 (73.8)	
1	5645 (27.4)	3082 (16.8)	8727 (22.4)	
≥2	1048 (5.1)	430 (2.3)	1478 (3.8)	

NCD: non-communicable disease.

### Association between tobacco smoking and NCDs

In Table 3, the unadjusted logistic regression analysis (Model A) indicated that active tobacco smoking was strongly associated with NCD prevalence (OR=1.67; 95% CI: 1.59–1.75). After adjusting for age, gender, and BMI (Model B), the AOR attenuated considerably to 1.27 (95% CI: 1.19–1.35). Further adjustment for education level, marital status, alcohol drinking, daily sleep time, daily sedentary time, and kitchen fume exposure (Model C) did not significantly alter the estimate, yielding an AOR of 1.26 (95% CI: 1.18–1.35). Furthermore, a dose-response relationship was evident for years of tobacco smoking. Compared to individuals who had smoked for <5 years, the AORs for NCDs were 1.24 (95% CI: 1.08–1.43) for those smoked for 5–14 years, 1.23 (95% CI: 1.09–1.39) for 15–24 years, and 1.37 (95% CI: 1.19–1.58) for those with ≥25 years of tobacco smoking (Model C). Daily cigarette consumption of ≥20 cigarettes was associated with a modest statistically significant elevation in risk compared to <20 cigarettes/day (Model C: AOR=1.13; 95% CI: 1.03–1.25). The effect size was larger in the unadjusted model (OR=1.31; 95% CI: 1.19–1.43) and reduced but remained statistically significant after adjusting for core demographics (Model B: AOR=1.16; 95% CI: 1.05–1.28).

**Table 3:** Association between tobacco smoking and NCD prevalence among residents undergoing physical examination in Changzhi, Shanxi Province, China, January–December 2025 (N=38906)

*Variables*	*Model A*		*p*	*Model B*		*p*	*Model C*		*p*
	*OR*	*95% CI*		*AOR*	*95% CI*		*AOR*	*95 % CI*	
**Tobacco smoking**									
Yes	1.67	1.59–1.75	0.000	1.27	1.19–1.35	0.000	1.26	1.18–1.35	0.000
No (ref.)	1			1			1		
**Secondhand smoke exposure**									
Yes	1.25	1.16–1.35	0.000	1.11	1.02–1.21	0.017	1.04	0.95–1.13	0.415
No (ref.)	1			1			1		
**Daily cigarette consumption**									
≥20	1.31	1.19–1.43	0.000	1.16	1.05–1.28	0.004	1.13	1.03–1.25	0.014
<20 (ref.)	1			1			1		
**Years of tobacco smoking**									
<5 (ref.)	1			1			1		
5–14	1.64	1.45–1.85	0.000	1.21	1.06–1.39	0.006	1.24	1.08–1.43	0.002
15–24	2.12	1.91–2.35	0.000	1.23	1.10–1.38	0.000	1.23	1.09–1.39	0.001
≥25	3.67	3.29–4.16	0.000	1.39	1.21–1.59	0.000	1.37	1.19–1.58	0.000

AOR: adjusted odds ratio. Model A: unadjusted model. Model B: adjusted for age, gender and BMI. Model C: adjusted as for Model B plus education level, marriage status, alcohol drinking, daily sleep time, daily sedative time, and kitchen fume exposure.

Regarding secondhand smoke, the crude association was significant (Model A: OR=1.25; 95% CI: 1.16–1.35). However, after adjustment for age, gender, and BMI, the AOR decreased to 1.11 (95% CI: 1.02–1.21). In the fully adjusted Model C, the association attenuated further and lost statistical significance (AOR=1.04; 95% CI: 0.95–1.13).

### Subgroup analysis for the association between smoking and NCDs

**Figure 2:**
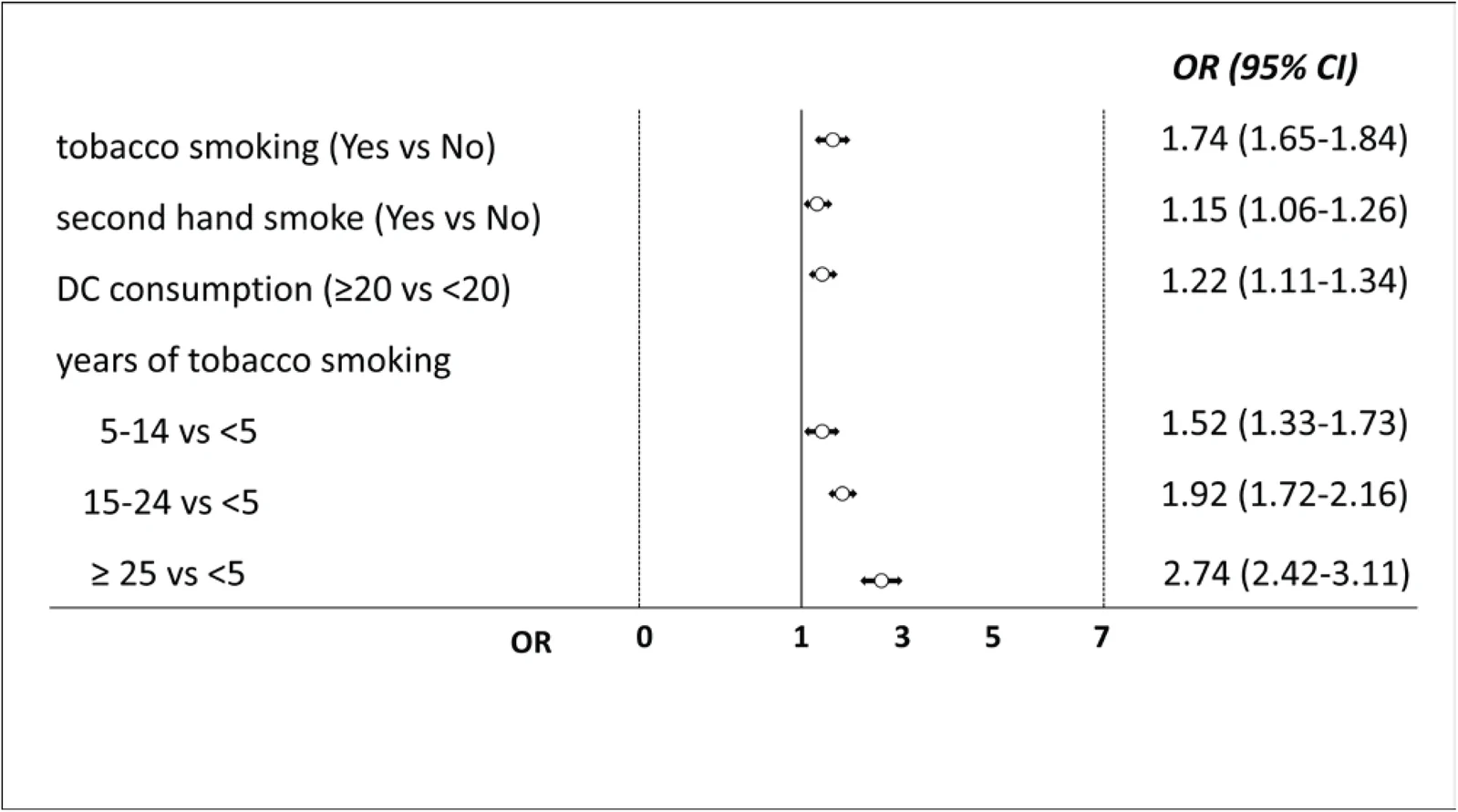
Association between tobacco smoke exposure and hypertension among residents undergoing physical examination in Changzhi, Shanxi Province, China, January–December 2025 (N=38906)

**Figure 3:**
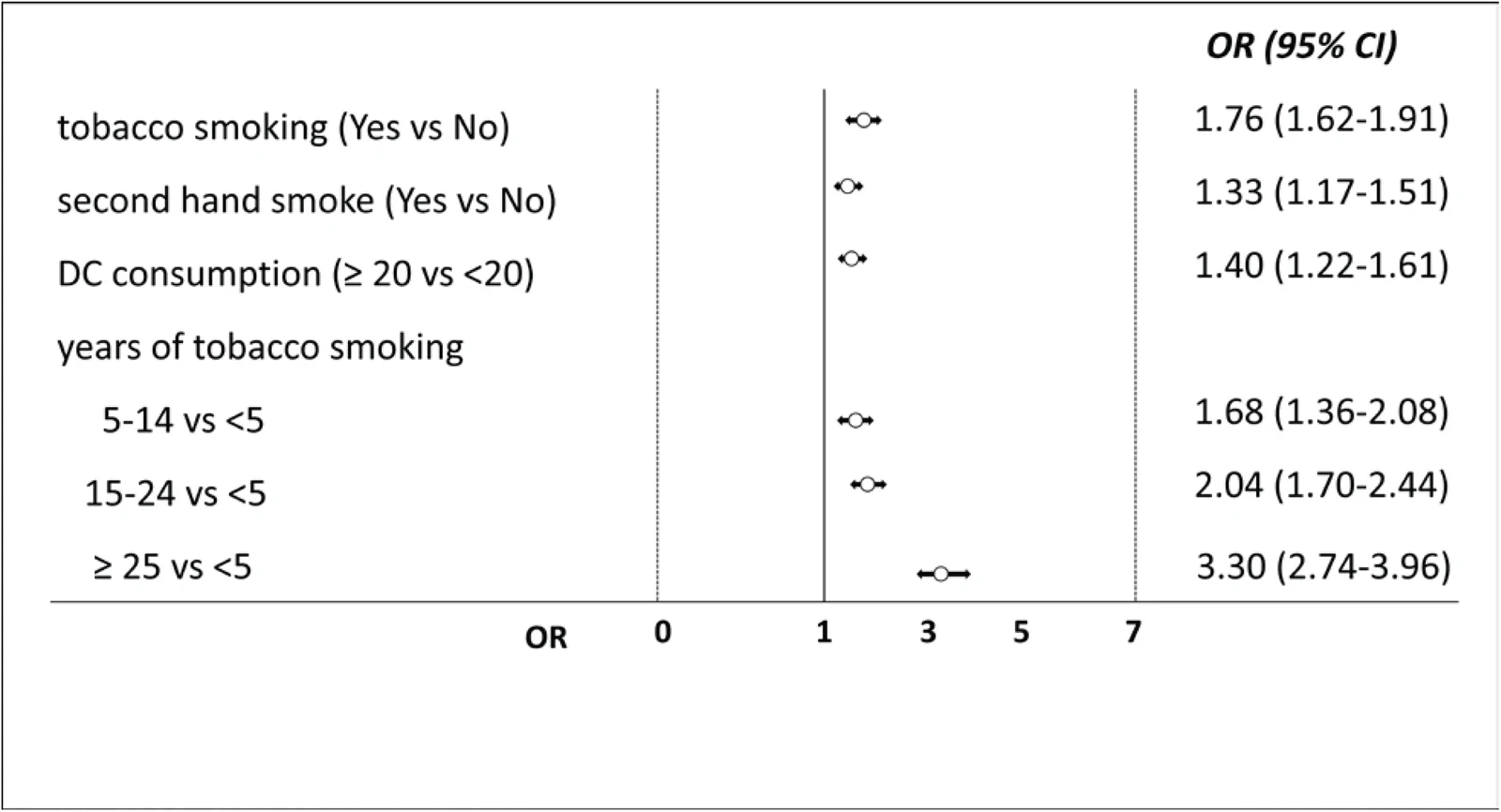
Association between tobacco smoke exposure and diabetes among residents undergoing physical examination in Changzhi, Shanxi Province, China, January–December 2025 (N=38906)

**Figure 4:**
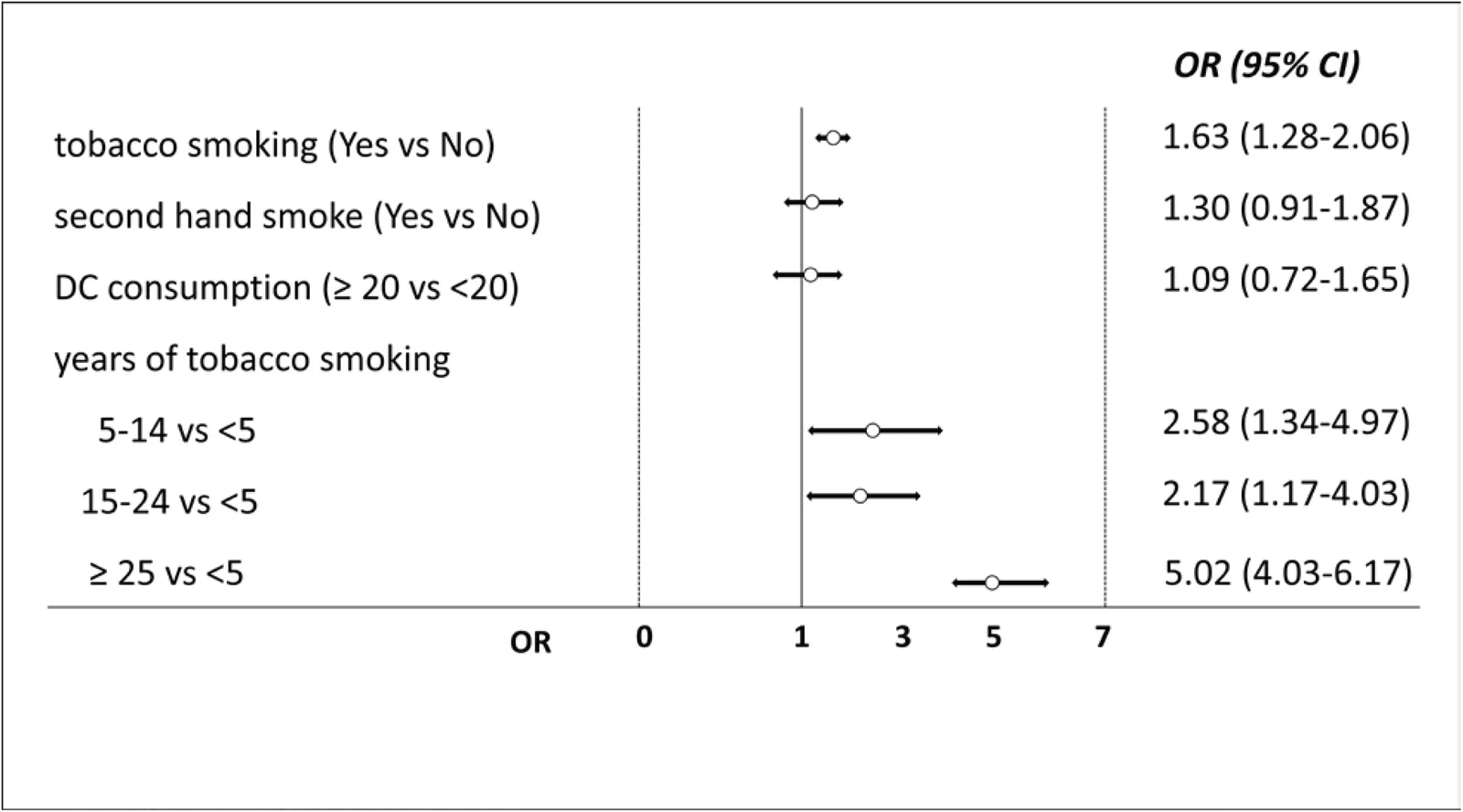
Association between tobacco smoke exposure and cardiovascular diseases in residents undergoing physical examination in Changzhi, Shanxi Province, China, January–December 2025 (N=38906)

**Figure 5:**
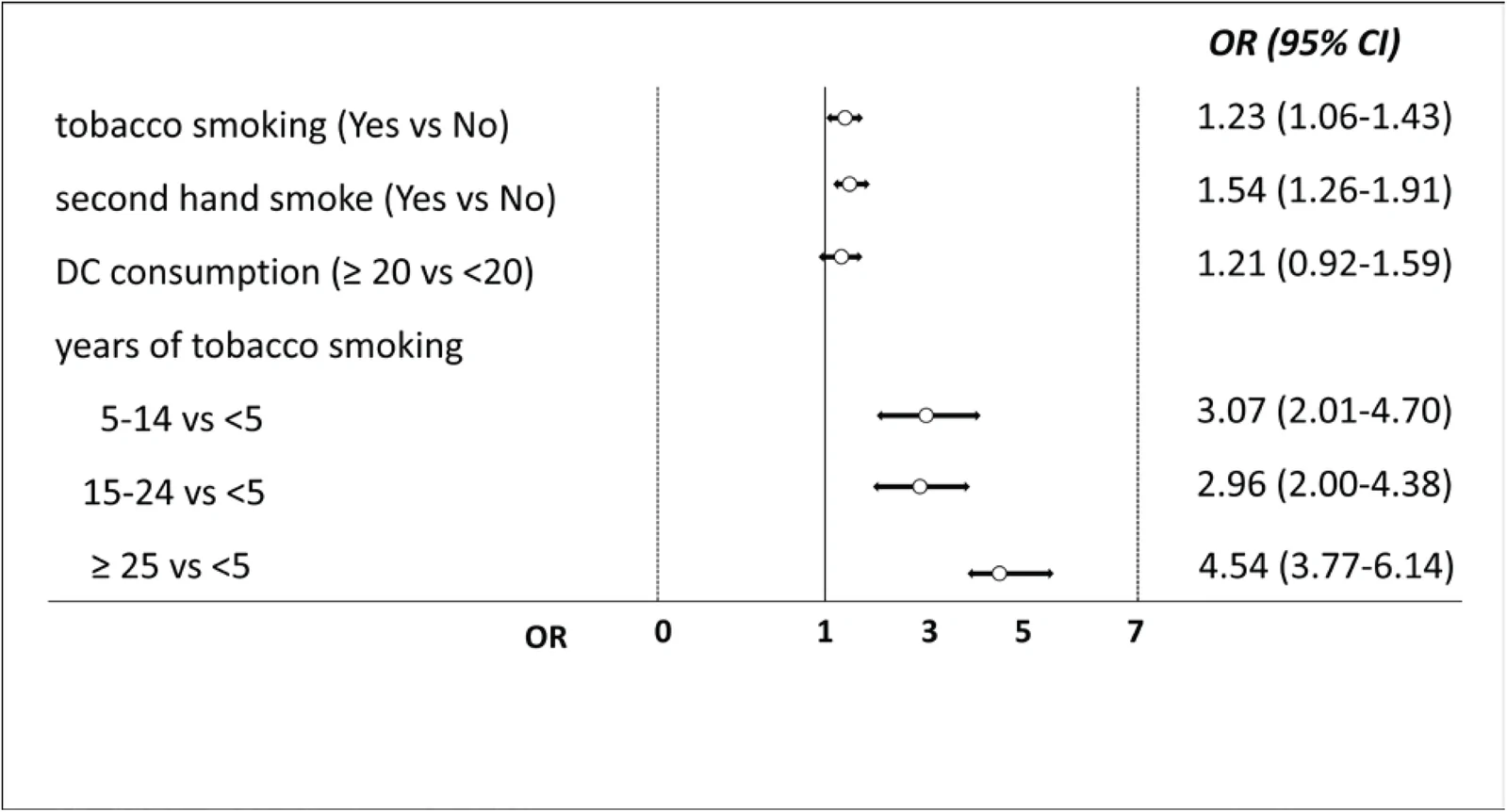
Association between tobacco smoke exposure and dyslipidemia among residents undergoing physical examination in Changzhi, Shanxi Province, China, January–December 2025 (N=38906)

The associations between tobacco smoke exposure and each individual NCD component of hypertension, diabetes, cardiovascular diseases, and dyslipidemia are depicted in Figures 2–5. Active smoking was significantly associated with hypertension (OR=1.74; 95% CI: 1.65–1.84), cardiovascular disease (OR=1.63; 95% CI: 1.28–2.06), diabetes (OR=1.76; 95% CI: 1.62–1.91), and dyslipidemia (OR=1.23; 95% CI: 1.06–1.43). Secondhand smoke showed weaker but significant associations with hypertension (OR=1.15; 95% CI: 1.06–1.26), diabetes (OR=1.33; 95% CI: 1.17–1.51), and dyslipidemia (OR=1.54; 95% CI: 1.26–1.91). High daily cigarette consumption (≥20 vs <20) was linked to hypertension (OR=1.22; 95% CI: 1.11–1.34) and diabetes (OR=1.40; 95% CI: 1.22–1.61). A progressive increase in odds with longer smoking duration was observed across all conditions, with ≥25 years of smoking conferring the highest risks: ORs of 2.74 (95% CI: 2.42–3.11) for hypertension, 5.02 (95% CI: 4.03–6.17) for cardiovascular disease, 3.30 (95% CI: 2.74–3.96) for diabetes, and 4.54 (95% CI: 3.77–6.14) for dyslipidemia.

## DISCUSSION

In this large cross-sectional study of health check-up attendees, we found that active tobacco smoking was an independent risk factor for the composite outcome of NCDs, with 26% excess odds after comprehensive adjustment. Importantly, a dose-response association was observed according to the duration of smoking, underscoring that the cumulative years of exposure, rather than merely daily intensity, serve as an important determinant of NCD prevalence. The association between secondhand smoke exposure and overall NCDs was no longer statistically significant after adjusting for potential confounders, highlighting the complexities inherent in isolating passive smoking effects in observational studies.

Active smoking was found to increase the odds of NCDs, confirming an extensive body of literature linking tobacco smoking to hypertension, diabetes, cardiovascular disease, and dyslipidemia^17-20^. Inhaled tobacco smoke delivers nicotine, carbon monoxide, and thousands of pro-oxidative and pro-inflammatory compounds. These toxicants induce endothelial dysfunction via reduced nitric oxide bioavailability, promote a state of chronic low-grade inflammation, impair pancreatic beta-cell function and insulin signaling, and alter lipid metabolism, thereby simultaneously fostering the development of multiple cardio-metabolic conditions^6,21-23^. Our use of a composite NCD outcome captures this systemic biological damage, which often results in multimorbidity – a hallmark of aging populations and heavy smokers. Compared with previous studies reporting 1.5- to 2.5-fold risks for individual diseases, the 1.26-fold increase observed in our fully adjusted model for any NCD reflects the conservative effect of comprehensive covariate control and the inclusion of a relatively healthy health check-up population with a lower background disease prevalence.

A particularly noteworthy finding was the dose-response pattern for smoking duration. The odds of NCDs were similarly elevated for those smoking 5–14 years and 15–24 years relative to very short-term smokers, but rose to 1.37 for those smoking ≥25 years. This suggests that for clinically manifested NCDs, a threshold of cumulative tobacco smoke exposure may need to be surpassed before a marked acceleration in risk occurs. This observation aligns with the ‘pack-years’ paradigm, which has been validated in cardiovascular and cancer epidemiology^24^. The relatively modest effect of daily consumption of ≥20 cigarettes compared with <20 cigarettes, further indicates that tobacco smoking intensity, as captured by current daily cigarette count, may have a ceiling effect, or that individuals may under-report the number of cigarettes smoked. It is also possible that in this study population, tobacco smoking duration was more strongly associated with disease because NCDs themselves develop over long latency periods, making cumulative exposure more relevant. Moreover, heavy smokers tend to die earlier and may be underrepresented in a health check-up sample, which would also attenuate the intensity-risk gradient through survival bias.

The secondhand smoke findings merit careful interpretation. Although SHS was significantly associated with NCDs in unadjusted models and showed strong links to hypertension, diabetes, and dyslipidemia, the association disappeared after full adjustment. Several factors may explain this. First, SHS exposure was relatively infrequent in our population (9.1%) and heavily skewed by gender, with the vast majority of exposed participants being non-smoking women married to smoking men. This introduces substantial collinearity with gender, marital status, and other socioeconomic variables. Second, we included kitchen fume exposure as a covariate, a unique and important indoor air pollutant predominantly affecting women. Since kitchen fumes and SHS share common correlates (e.g. being a non-smoking female in a household with a smoker), their simultaneous inclusion may have led to overadjustment or partitioning of the effect, making it difficult to detect an independent SHS association. Third, misclassification of SHS exposure is likely, as self-report may not accurately reflect biological uptake; cotinine measurements would be preferable but were unavailable. Importantly, the lack of statistical significance in the fully adjusted model does not negate the established causal relationship between SHS and cardiovascular and respiratory disease, nor does it imply that SHS is harmless. Instead, it underscores the challenge of disentangling low-level, pervasive environmental exposures from strongly correlated lifestyle factors in a single cross-sectional study. Public health policies mandating smoke-free environments remain critical for primary prevention.

The associations between active tobacco smoking and specific NCDs are consistent with global estimates. The stronger effect sizes for hypertension, cardiovascular disease and diabetes likely reflect the substantial contribution of tobacco smoking to vascular stiffness, sympathetic activation, insulin resistance, and visceral adiposity. The smaller OR for dyslipidemia may be due to competing mechanisms: while tobacco smoking clearly promotes atherogenic lipid profiles (higher triglycerides, lower HDL cholesterol)^25-29^, some smokers may maintain relatively normal lipid levels through still-unclear compensatory pathways or because dyslipidemia in this study population was based on clinical diagnosis rather than a rigorous laboratory cutoff. Additionally, the low overall prevalence of diagnosed dyslipidemia (2.0%) suggests underdiagnosis, which would also bias the OR toward the null.

### Strengths and limitations

This study has several notable strengths. With nearly 39000 participants, it is one of the largest single health-check-up studies to evaluate the smoking–NCD association in China. We incorporated a wide array of covariates rarely simultaneously collected, including kitchen fume exposure, daily sleep time, and sedentary behavior, which are important confounders in the modern lifestyle context. The use of standardized physical examinations allowed precise measurement of BMI, waist circumference, and other anthropometric parameters. By analyzing a composite NCD outcome, we directly address the emerging clinical reality of multimorbidity.

Nevertheless, several limitations warrant discussion. The cross-sectional design prohibits causal inference; individuals with NCDs may have reduced smoking, quit smoking, or avoided tobacco exposure following diagnosis, potentially biasing the OR for current smoking toward the null. Disease diagnoses were based on self-reported medical history and health examination records, which may be subject to recall bias and misclassification, particularly for dyslipidemia and early-stage diabetes. Our sample consisted of health check-up participants, who are generally more health-conscious and have better access to medical care than the general population, limiting external validity. The extremely low prevalence of female smoking (0.3%) and sexual dimorphism in exposure patterns (men dominated active smoking, women dominated kitchen fume exposure) made it impossible to stratify effectively by gender, and residual confounding by sex cannot be excluded. Although we adjusted for a wide range of lifestyle factors, unmeasured confounders such as dietary quality, physical activity intensity, and genetic susceptibility were not accounted for. Finally, SHS exposure was based on a single binary question, which likely led to exposure misclassification; future studies would benefit from biochemical verification.

From a clinical and public health perspective, our findings reinforce the importance of prioritizing smoking cessation interventions for long-term smokers, as cumulative smoking years were more strongly associated with NCD prevalence than current daily cigarette consumption. Even reductions in the number of cigarettes smoked per day may confer limited benefit if total smoking duration remains prolonged, though complete cessation unequivocally lowers risk. Comprehensive tobacco control policies including tobacco taxation, advertising bans, and smoke-free legislation should continue to be implemented. For clinicians, incorporating lifetime smoking history and pack-years into cardiovascular risk assessment tools, may improve risk stratification. Furthermore, our data highlight that women in this study population, despite very low active smoking rates, carry a considerable burden of NCDs (19.2%), likely related to secondhand smoke exposure, kitchen fumes exposure, and metabolic factors. Addressing these multiple environmental and behavioral risks in an integrated manner is important to curbing the NCD epidemic.

## CONCLUSIONS

Active tobacco smoking is an important risk factor for prevalent NCDs among health check-up populations, with a potential cumulative effect related to smoking duration. The absence of an independent association for SHS after comprehensive adjustment reflects the complex interplay of correlated environmental and lifestyle risk factors but does not diminish the imperative to eliminate all forms of tobacco smoke exposure. Thus, tobacco control strategies and targeted cessation programs for long-term smokers are essential to reduce the growing NCD burden.

## Data Availability

The data supporting this research are available from the authors on reasonable request. The request should state the title and aim of the research for which the data are requested.
